# A Methodological Perspective on the Function and Assessment of Peripheral Chemoreceptors in Heart Failure: A Review of Data from Clinical Trials

**DOI:** 10.3390/biom12121758

**Published:** 2022-11-26

**Authors:** Maksym Jura, Mateusz Garus, Kornelia Krakowska, Szymon Urban, Mikołaj Błaziak, Gracjan Iwanek, Robert Zymliński, Jan Biegus, Bartłomiej Paleczny

**Affiliations:** 1 Department of Physiology and Pathophysiology, Wroclaw Medical University, 50-376 Wroclaw, Poland; 2 Institute of Heart Diseases, Wroclaw Medical University, 50-556 Wroclaw, Poland; 3 Department of Nephrology and Transplantation Medicine, Wroclaw Medical University, 50-556 Wroclaw, Poland

**Keywords:** peripheral chemoreceptors, heart failure, cardiology, pathophysiology

## Abstract

Augmented peripheral chemoreceptor sensitivity (PChS) is a common feature of many sympathetically mediated diseases, among others, and it is an important mechanism of the pathophysiology of heart failure (HF). It is related not only to the greater severity of symptoms, especially to dyspnea and lower exercise tolerance but also to a greater prevalence of complications and poor prognosis. The causes, mechanisms, and impact of the enhanced activity of peripheral chemoreceptors (PChR) in the HF population are subject to intense research. Several methodologies have been established and utilized to assess the PChR function. Each of them presents certain advantages and limitations. Furthermore, numerous factors could influence and modulate the response from PChR in studied subjects. Nevertheless, even with the impressive number of studies conducted in this field, there are still some gaps in knowledge that require further research. We performed a review of all clinical trials in HF human patients, in which the function of PChR was evaluated. This review provides an extensive synthesis of studies evaluating PChR function in the HF human population, including methods used, factors potentially influencing the results, and predictors of increased PChS.

## 1. Introduction

Peripheral chemoreceptors (PChR) are essential oxygen sensors in the human body, crucial for maintaining proper oxygen (O_2_), carbon dioxide (CO_2_), and hydrogen ions concentrations in the bloodstream [1,2,3]. Their physiological role is in preventing hypoxemia and optimizing the O_2_ supply to organs [4] by activating the rapid systemic responses, including ventilatory and arterial pressure augmentations [3,4]. The stimulation of PChR elicits sympathetic activation, whereby its function influences autonomic balance [5,6,7,8]. PChR overactivity is a hallmark of various sympathetically mediated diseases [9,10], for example, heart failure (HF) [11,12], hypertension [13,14], obstructive sleep apnea [15,16,17,18] and metabolic abnormalities [19,20], and is suspected to be an important driver of sympathetic hyperactivity in these disorders [10,11,18]. HF is a disabling clinical syndrome causing a growing number of hospital admissions in recent years [21,22], that manifests as an increased sensitivity of the PChR [23,24,25] (PChS) represented by exaggerated hypoxia-triggered increases in sympathetically mediated ventilation and hemodynamic responses [26], as well as augmented tonic PChR activity [9,10], manifested by an enhanced decrease in ventilation and sympathetic activity after the inhibition of PChR [27,28,29]. The magnitude of PChS correlates with the severity of HF [23,24,30,31]. The overactivity of PChR is related to an enhanced prevalence of supraventricular and ventricular arrhythmias [23,24], the ventilatory response to exercise [24,32,33], and abnormal patterns of breathing [34]. Moreover, increased PChS is a well-established independent predictor of a poor prognosis in HF [25,35,36]. The role of PChR in the progression and prognosis of HF, the causes of their distorted function, and possible modulation methods is the subject of intense research. In recent times, many valuable reviews in these matters have been published [4,11,37,38,39,40,41], however, none of them focuses on data from all clinical trials evaluating both PChS and tonic PChR activity in HF human patients, their methodology, results, and the potential impact of HF treatment.

## 2. Physiology and Pathophysiology of Peripheral Chemoreceptors

**Figure 1:**
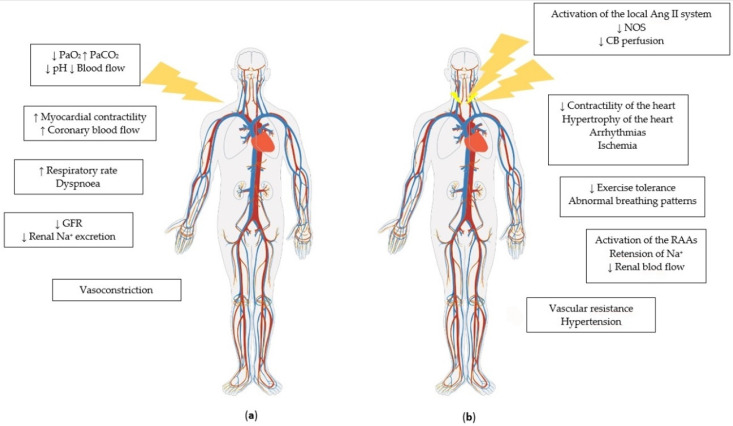
(**a**) Illustration of the triggers and organic effects of carotid body activation; and (**b**) Possible causes of carotid body overactivity and its organic complications. Ang II—angiotensin II; CB—carotid body; GFR—glomerular filtration rate; Na^+^—natrium ion; NOS—nitric oxide synthase; PaCO_2_—partial pressure of carbon dioxide; PaO_2_—partial pressure of oxygen; pH—potential of hydrogen.

The dominant PChR are carotid bodies (CBs) located at the bifurcation of each common carotid [10]. CBs are innerved by the carotid sinus nerve, vagal nerve, and sympathetic nerve of the superior cervical ganglion [10]. PChR are predominantly sensitive to hypoxia [42] but are also sensitive to acidosis, hypercapnia, hyperthermia, hypoosmolarity, hyperglycemia, inorganic phosphate, sodium cyanide, and hypoperfusion (Figure 1) [9,10,43]. The activation of PChR mediates sympathoactivation causing an increase in blood pressure and minute ventilation (MV) [2] and also causing an inhibition of the baroreflex function [29,44]. The direct stimulation of CBs with adenosine causes a decrease in heart rate [45], however, the activation of PChR with hypoxia manifests tachycardia [31]. Based on animal studies, the response from the CBs was believed to include primary and secondary reflexes [46,47]. The primary response includes bradycardia and vasoconstriction. The secondary reflex contains tachycardia and vasodilatation caused by hyperventilation, activating a reflex from the pulmonary stretch receptor (Hering–Breuer reflex), and depends on the magnitude of the increase in ventilation [9]. However, some studies have indicated that hypoxic tachycardia is not secondary to hyperpnoea [48,49,50,51]. The most probable explanation seems to be that hypoxic tachycardia is mediated by aortic chemoreceptors [52,53], another cluster of PChR besides CBs.

Sympathetic hyperactivity is associated not only with the development of the disease but also with its progression and poor prognosis. The increased sympathetic drive can lead to numerous pathological mechanisms, i.a., hypertrophy of the heart, arrhythmias, ischemia, vasoconstriction, the release of renin and sodium retention in the kidney and increased renal vascular resistance reducing renal blood flow (components of the cardiorenal syndrome) [10,54]. The increases in vascular resistance and blood volume increase preload and afterload, and, consequently, cardiac work for the damaged myocardium [54]. The increase in cardiac sympathetic nerve activity is linked to abnormal calcium cycling and calcium leakage in the failing myocardium, which promotes a decrease in myocardial contractility [55,56].

Possible mechanisms of augmented PChS in HF are subject to intense research. Studies conducted mostly on animal models indicated some potential mechanisms, such as the activation of the local angiotensin II system [57,58], decreased levels of nitric oxide synthase (NOS) [59,60], or reduced perfusion of CBs [61]. As we mentioned above, CBs not only modulate sympathetic activation but also receive innervation from both sympathetic and parasympathetic systems. Animal studies revealed that the efferent impulsation in these nerves, specifically sympathetic stimulation, influences PChS [62,63], which could be caused by direct stimulation or vasoconstriction causing the hypoperfusion of CBs.

PChR function includes PChS (phase activity) and tonic activity [9,10], which can be aroused during normoxic breathing [13,64,65]. Increased PChR phase and tonic activity can directly lead to sympathetic overactivity and baroreceptor dysfunction [13,66]. What is interesting is that these two aspects of the PChR function do not have to always be associated [10] as Paton et al. proved by presenting the case of a hypertensive patient with low PChS but increased PChR tonicity [9]. That issue remains to be profoundly investigated, as the patients with elevated PChR tonic activity, undetected by classic acute hypoxic methods, are in danger of developing complications from sympathetic overactivity leading to the progression of their disease.

The methods of PChR assessment can be divided into methods of assessing their tonic activity, in which we deactivate chemoreflex using hyperoxia or low-dose dopamine, and assessing their phase activity, in which chemoreceptors are stimulated by a decrease in blood oxygen saturation.

In our work, we aimed to present and summarize studies conducted on HF patients concerning the phase or tonic activity of PChR, published in English before May 2022. We compared the methods of its assessment, characteristics of studied populations, and potential clinical predictors of augmented chemosensitivity. Finally, we managed to identify 22 studies meeting our criteria (13 assessing only phase activity [23,24,25,31,32,34,35,36,67,68,69,70,71], 4 assessing both phase and tonic activity [30,33,72,73], and 5 assessing only tonic activity [27,28,29,74,75]).

## 3. Methods of Assessment of PChR Phase Activity

In studies assessing phase activity (Table 1), the overwhelming majority (13 of 17) used a transient hypoxia test with pure nitrogen (N_2_) to determine the phase activity of PChR [23,25,30,31,32,33,34,67,68,69,70,72,73]. This method involves repeated transient exposures to N_2_ distributed into the breathing circuit to obtain a wide range of minimal saturations (usually between 70–90%). These nadirs of saturation are plotted against the maximal MV recorded for each exposure. That dependence is expressed as the slope of the regression line that defines the magnitude of PChS [25]. PChS that exceeds the mean value from a healthy population + 2 standard deviations (SD) is considered augmented [23]. The second most used method was a rebreathing technique (isocapnic progressive hypoxic method) which was harnessed in four studies [24,35,36,71]. This technique requires the usage of a closed circuit with a 5–6 L bag connected to the patient through a two-way non-rebreathing valve. To prevent the activation of central chemoreceptors, end-tidal CO_2_ is held constant by a CO_2_-absorbing bypass, through which a portion of the expired air is passed before returning to the bag [36,76].

Both mentioned methods use hypoxia to stimulate PChR. Systemic hypoxia, however, is not the pure PChR activator, due to its hyperpolarizing effect on the vascular smooth muscle, which could cause a decrease in blood pressure, which in turn could activate the response from baroreceptors, which is antagonistic to chemoreflex [9].

**Figure 2:**
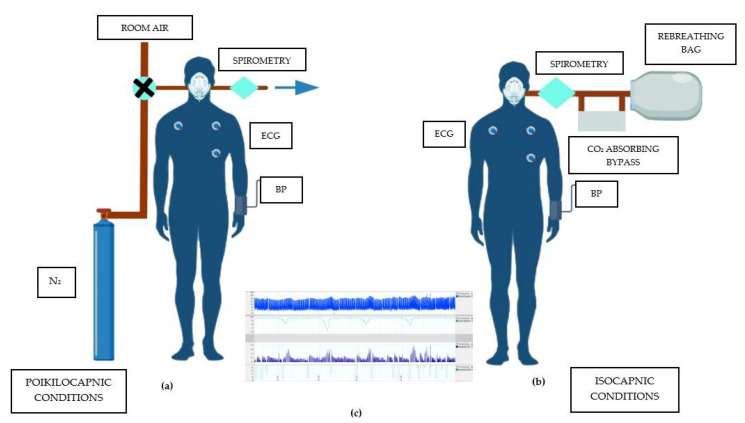
(**a**) Illustration of the transient hypoxia method; (**b**) Illustration of the isocapnic progressive hypoxic method (rebreathing technique); and (**c**) Example of the measurement of peripheral chemosensitivity. For each nadir of saturation minute ventilation, heart rate and systolic blood pressure values are plotted. BP—blood pressure; CO_2_—carbon dioxide; ECG—electrocardiography; and N_2_—nitrogen.

The main difference between these two methods (Figure 2) is carbon dioxide partial pressure (PCO_2_). The transient hypoxia method is conducted in poikilocapnic conditions. Changes in PCO_2_ may alter the concentration of hydrogen ions that can modulate the function of peripheral and central chemoreceptors [77] and the hypocapnia caused by blowing off CO_2_ during hyperventilation accompanying long N_2_ deliveries is capable of impairing HVR which can be the cause of the underestimation of PChS [38]. On the other hand, hypercapnia can exaggerate HVR [38,39]; that is why it could be crucial to maintain isocapnic conditions during the test. Constant CO_2_ levels could exclude the activation or inhibition of central chemoreceptors and the modulation of hypoxic response from PChR [39], but another potentially problematic aspect is the proper choice of isocapnic PCO_2_. As shown by Keir et al., during an experiment with a transient hypoxia test under different isocapnic conditions and poikilocapnic conditions in one subject, the greater the levels of isocapnia, the PChS raises [38]. Moreover, with a higher end-tidal PCO_2_, the regression lines are shifted upward, which is the result of the activation of central chemoreceptors [38]. The transient hypoxia method with its poikilocapnic conditions can cause an underestimation of exaggerated PChS, on the other hand, titrating CO_2_ to maintain isocapnic conditions during the progressive hypoxic isocapnic method could be technically problematic and the selection of proper PCO_2_ remains not without significance.

The majority of data on PChR function in various disorders is derived from the assessment of the ventilatory component of the chemoreflex only. Consequently, an augmented PChS is usually referred to the exaggerated HVR. Nevertheless, some of the recent studies performed on healthy subjects [78] and obstructive sleep apnea [79] patients have claimed that the ventilatory and sympathetic components of the peripheral chemoreflex are not related to each other in response to PChR stimulation and inhibition [78,79]. If HVR also does not predict sympathetic activation in the HF population, the usefulness of trials targeting only that response could be put into question [78]. Therefore, it may be necessary to employ a comprehensive method for the assessment of the PChS, including the ventilatory and neurocirculatory (hemodynamic and sympathetic) components of the chemoreflex.

## 4. Comparison of Studied Populations

Sixteen studies containing an assessment of the phase activity of PChR were conducted in Europe and one was conducted in Canada [73]. Included patients mostly had reduced left ventricular ejection fraction (LVEF), except for two patients with preserved LVEF enrolled in the study by Collins et al. [73]. The publications’ dates extend to 26 years. During that time, knowledge about pathophysiology and HF treatment paradigms have changed diametrically. Noteworthy, despite those changes in approaches to HF pharmacotherapy, the prevalence of increased chemosensitivity has not changed considerably. In 1997, it amounted to 40% (mean PChS: 0.673 ± 0.41 L·min^−1^·%SpO_2_^−1^) [23] and 42% (0.72 ± 0.36 L·min^−1^·%SpO_2_^−1^) [30] versus 44% (0.58 [0.32–0.95] L·min^−1^·%SpO_2_^−1^) [31] and 40% (0.6 ± 0.4 L·min^−1^·%SpO2^−1^) [72] in 2013 and 2021, respectively (all studies used the same method). That fact appears surprising in view of the knowledge of the possible pharmacological impact on PChR [40].

## 5. The Impact of HF Etiology and Management on PChR Function

### 5.1. Digoxin

Digoxin was used by a considerable percentage of patients in the first studies and was eventually replaced by beta-blockers (BBs). However, that change has neither altered the mean PChS nor the prevalence of increased PChS. This is despite the fact that digoxin possesses a proven impact on autonomic balance. Digoxin augmented the baroreceptor sensitivity in healthy subjects and HF patients [80]. In healthy humans, digitalis enhances HVR with no alteration in the HCVR [81,82]. In HF patients, digoxin abolished the typical hemodynamic reaction to hyperoxia [83].

### 5.2. Angiotensin-Converting-Enzyme Inhibitors

In all revised publications, the majority of studied HF patients have taken angiotensin-converting enzyme inhibitors or angiotensin receptor blockers, so their impact on PChS cannot be compared. Despite their influence on PChS not having been tested in animal or human subjects, there are only a few possible pathways for their modulation of PChS since, in animal models of HF, the overexpression of angiotensin receptors in carotid bodies has been found [84,85]. Moreover, the administration of angiotensin II augmented PChR activity and PChR response to hypoxia in animal models [85,86]. On the other hand, Brown et al. claimed that the blockade of angiotensin II type I receptor with losartan did not influence HCVR, although pure HVR was not evaluated [87].

### 5.3. Beta-Blockers

In light of knowledge about the role of adrenergic drugs in the modulation of PChR response, the results presented by Ponikowski et al. seem to be interesting [67]. In that study, none of the participants were treated with BBs and 64% of patients were reported with a higher PChS compared to healthy controls from the same team’s previous research with the same utilized method [32,88]. However, the mean PChS among these subjects was not significantly different from the HF patients treated with BBs in the study utilizing a likewise method [72]. That is surprising because other studies have shown the impact of certain BBs on a decrease in resting ventilation (during normoxia and hypoxia) and exercise ventilation [89] and their capability to reduce PChS [90]. That ability could be explained by the protein expression of β1 and β2-adrenoceptor subtypes in type I cells in carotid bodies [91]. Nebivolol could also modulate PChS via the nitric oxide (NO) pathway. Nerves and vessels surrounding type I cells of the carotid body contain the enzyme NOS [92,93], the reduction in the expression of which was demonstrated in the HF animal model [94], and the NOS inhibitor enhanced the sympathoexcitatory response to hypoxia [94]. That may suggest that a deficiency of NO in the carotid bodies in HF augments PChS. A Beta-blockade has also proven the ability to increase baroreflex sensitivity, which is an antagonist to PChS [95,96], although that blockade did not alter the predictive value of baroreflex sensitivity [97].

### 5.4. Mineralocorticoid Receptor Antagonists

Mineralocorticoid receptor antagonists (MRA), as a part of HF therapy, were reported for the first time in the study of PChR in the work by Giannoni et al. from 2008 [24] and became an integral component of HF therapy in later-revised studies. The impact of MRA intake on PChS has not been contributed, although, in healthy humans, aldosterone impaired the baroreflex response [98], while aldosterone antagonists improved cardiac vagal control [99]. The antagonistic function of baroreflex and chemoreflex suggests that MRA could also have an impact on PChS. Another possible mechanism of MRA’s impact on PChR is the ability to reduce sympathetic nervous system activity, in which overactivation can stimulate PChR [100], although the introduction of MRA in HF treatment guidelines has not significantly changed the prevalence of increased PChS.

### 5.5. Diuretics

In all revised papers a significant number of patients received diuretic therapy, whereby in older studies, this percentage was around 100% and decreased with time to 50–70%. The impact of usually prescribed groups of diuretics: loop, thiazide, and thiazide-like, on PChR has not been studied. The only diuretic drug with a proven impact on PChS is carbonic anhydrase inhibitor—acetazolamide [40]. Unfortunately, not every study specifies the type of diuretic that patients received, and those which provide that information do not mention acetazolamide.

### 5.6. Antiplatelet Drugs

There is no information available in the publications, so far, concerning antiplatelet therapies in the studied population. Antiplatelet drug—P2Y12 inhibitor—ticagrelor increases adenosine tissue concentrations [101]. The stimulating effect of the intracarotid administration of adenosine on the activity of PChR was reported in animal [102] and human studies [45]. Moreover, its impact on central chemosensitivity was proven [103], although it needs further evaluation.

### 5.7. Statins

Unfortunately, none of the publications provide information about lipid-lowering treatment. Because the majority of studied patients presented HF secondary to the ischemic cause, a considerable part of them were probably treated with statins. Statins have a proven impact on the modulation of PChS. A possible mechanism of their influence is the induction of a Krüppel-like factor 2 (KLF2) expression in the CB cells. KLF2 is a transcription factor that regulates the expression of enzymes associated with NO bioavailability, angiotensin metabolism, antioxidant defenses, and inflammation [104]. These enzymes play a role in increased PChS. In HF, KLF2 expression is reduced in CBs which is associated with increased PChS [105]. Treatment with statins was associated with increased KLF2 expression in CBs as well as with a decrease in PChS [105,106,107]. KLF2 is probably mediating the statin’s ability to increase the accumulation of NOS in endothelial cells [106,108,109].

### 5.8. Devices

In three revised studies, patients were treated with device therapy [31,35,36], which, from a physiological point of view, could have a potential impact on PChS. It has been demonstrated that cardiac resynchronization therapy (CRT) reduces muscle sympathetic nerve activity (MSNA) in the responders to CRT [110], and increases baroreflex sensitivity [111]. Nevertheless, in mentioned studies, no significant differences in PChS were reported between patients with or without implantable devices. That could be the result of a relatively small percentage of patients with CRT in studied populations as well as not dividing them into responders and nonresponders to CRT.

### 5.9. Etiology

The majority of studied patients had developed HF due to ischemic heart disease (IHD), and only in one study was the prevalence of idiopathic dilated cardiomyopathy higher than IHD [24]. None of the authors reported significant differences in PChS regarding etiology. That is interesting, taking into consideration that in ischemic HF, sympathetic activation is higher than in non-ischemic HF, when compared [112,113], probably due to the chronic stimulation of sympathetic afferent nerve endings in the anterior and inferoposterior walls [114] of the ischemic heart [115]. Another possible explanation is the impairment of the ventricular mechanoreceptor input to vagal afferents caused by ischemic injury [116]. A greater density of receptors on vagal afferents was found in the inferoposterior wall [116]. The ability of ischemia to both trigger or inhibit cardiac reflexes and differences in the distribution of autonomic afferent nerves in the heart elicits the necessity of evaluating not only the etiology but also the location of lesions when assessing PChS.

## 6. Methods of Assessment of PChR Tonic Activity

In nine studies, the tonic activity of PChR was assessed. In six of them, acute hyperoxia with 100% oxygen was used [29,30,33,72,74,75] (Table 2), meanwhile, two studies used low-dose dopamine [28,73]. In one study both methods were applied [27].

Hyperoxia temporarily blocks chemosensory function, causing a decrease in sympathetic activity, which reflects the magnitude of tonic PChR activity. Dopamine blocks the release of neurotransmitters from the carotid body type I cells, inhibiting afferent signaling in the carotid sinus nerve [39]. It is not without significance that dopamine does not cross the blood-brain barrier, so central chemosensitivity remains unaffected [117,118]. However, both methods have some serious limitations. Acute hyperoxia, besides its inhibiting effect on PChR, also has a direct vasoconstriction effect [119,120], probably because of reactive oxygen species impairing the function of endothelium factors responsible for maintaining vascular tone [121,122,123]. For this reason, it could be complicated to distinguish the effects of PChR inhibition from those caused directly by oxygen. Low-dose dopamine infusion is also not free from side effects, including tachycardia and hypotension that may influence findings and interpretations [124]. Hemodynamic effects of low-dose dopamine infusion can arise both from the direct influence on dopamine receptors in peripheral vessels [125,126] and the inhibition of PChR [124]. A possible direct influence of dopamine on sinus node β-receptors [127] can be the interference of the inhibiting effect on PChR on indices of autonomic balance, such as heart rate variability (HRV) [124], hindering the interpretation of the results. An additional problem is the selection of an appropriate dose of dopamine. As shown by a study on healthy volunteers, there is a large intersubject variability in the range of low doses of dopamine inhibiting PChR [128]. The appropriate dose differed between subjects with high and low baseline chemosensitivity [128]. Moreover, some authors claim that mild hypercapnia caused by attenuated MV with increased end-tidal carbon dioxide values can stimulate central chemoreceptors causing an underestimation of ventilation attenuation on dopamine [128]. However, the dopamine method is the only one that enables a concomitant study of the phase activity of PChR by acute hypoxic response [124], which is not possible using the hyperoxia method. The optimal protocol seems to be the assessment of the PChR phase activity first, and then of tonic activity with the use of an appropriate dose of dopamine.

Authors of revised studies present different methodologies for the assessment of the impact of hyperoxia on PChR and autonomic balance. Chua et al. [33] analyzed the magnitude of the fall in MV; Ponikowski et al. investigated autonomic balance reflected as a spectral analysis of HRV [30]; Hennersdorf et al. divided the difference between the mean RR-interval before and after oxygen inhalation by the difference between venous partial oxygen pressure before and after oxygen inhalation [74,129]; Two papers presented by French researchers assessed sympathetic baroreflex function represented as the relationship between MSNA and diastolic blood pressure [29,75]; Edgell et al. [27], as well as Tubek et al. [72], investigated both ventilatory and hemodynamic responses to hyperoxia.

Establishing an optimal protocol for evaluating the tonic activity could be crucial in further investigation, as the patients with elevated tonic PChR activation could potentially be overlooked by classic hypoxic methods, whereas they could benefit from therapies targeted at restoring balance in the autonomic control of the cardiovascular system. In the literature, however, there is a greater prevalence of work evaluating PChR response to an acute hypoxic stimulus [38] than its tonic activity in the HF population. As mentioned above, these two aspects of the PChR function could not always be associated [9,10]. Patients with augmented CB tonicity could remain undetected by classic methods of assessing the PChR phase activity. As we also mentioned above, in methods using hyperoxia as well as using hypoxia, the ventilatory response does not solely predict a sympathetic response [79]. Because sympathetic overactivity seems to be the cause of the progression of HF and poor prognosis, the methods of assessment of PChR tonic function evaluating the decrease in MSNA in response to hyperoxia pretend to be more useful.

We managed to find three papers concerning the impact of dopamine on PChR in HF patients (Table 3). The authors did not divide patients according to PChS, and the dose of administrated dopamine did not differ between subjects with normal and augmented PChS. Although the usage of different doses, the results of the experiments were similar. The studies established the dopamine method as a feasible tool for the assessment of tonic activity in HF patients. What should be a matter of concern for further research is the appropriate dose selection according to patients’ PChS and the assessment of the ventilatory, hemodynamic, and sympathetic effects of dopamine infusion in order to reduce distorting effects of hipercapnicstimulation of central chemoreceptors.

## 7. Predictors of Increased Chemosensitivity

The assessment of the PChR function is a time-consuming, complex procedure, that could probably help us with the qualification of patients to the new forms of therapy consisting of autonomic balance modulation. To facilitate the selection of patients who should undergo such evaluation, we analyzed the available literature in terms of possible predictors of augmented chemosensitivity (Table 4).

## 8. Novel Therapies and Possibilities Research Gaps

Novel methods of HF therapy use knowledge of the role of the autonomic system in the progression and prognosis of HF and aim at pharmacological and non-pharmacological modulation of the neurohormonal system [44,130,131,132,133,134,135].

One of the proposed therapies in HF may be the inactivation of CBs [58,64,136]. Two studies with the removal of CBs in humans have recently been performed [12,137] and showed a significant reduction in the activity of the sympathetic nervous system (especially in the case of bilateral resection) and an improvement in the quality of life and exercise tolerance [12]. CBs resection may also prevent left ventricular remodeling and a reduction in LVEF, as well as life-threatening arrhythmias, which directly translate into survival [12,137]. As an open surgical procedure, CBs resection can cause complications of some kind [10], which should be eliminated through new noninvasive methods that are currently under clinical trials (ClinicalTrials.gov; NCT02099851, NCT03314012). Another, potentially dangerous complication that merits mention is the risk of significant oxygen desaturation [138]. Therefore, the qualification of patients for carotid body resection should be carried out with extreme caution and after considering the potential benefits and risks of complications. Using optimal protocols in the assessment of PChR function in patients with clinical predictors of distorted PChR function could be a useful tool in such a process.

An extremely interesting and promising form of therapy is baroreflex activation therapy (BAT), which uses afferent signaling to the central nervous system through the nerves of the carotid sinus, thus inhibiting the sympathetic system and stimulating the parasympathetic system to restore autonomic balance [139]. Several clinical trials have so far been conducted, which have demonstrated the efficacy (improvement in the quality of life, reduction in the frequency of readmissions) and safety of BAT [140,141].

It is necessary to mention the possibility of modulating the superior cervical ganglion, which directly affects the CBs [10]. However, in the available literature, attempts to interfere with this part of the nervous system are described only in animal models, without precise data on long-term effects [142].

Our work on this review enabled us to identify some research gaps in terms of PChR in HF populations. The majority of the available literature is concerned with stable patients with chronic HF, while no studies evaluating the function of the PChR among patients with acute heart failure (AHF) have been found. Patients hospitalized due to AHF are a special group of patients who can benefit from innovative forms of therapy based on neuromodulation. These patients, among others, suffer from dyspnea and hyperventilation (evidenced by hypocapnia), which might well be caused by the overactivity of PChR [143]. Currently, the assessment of carotid body chemosensitivity in patients with AHF associated with dyspnea and hypocapnia is being conducted in our center.

## 9. Limitations

Our study is not free from limitations. Importantly, this is a literature review and was not performed in accordance with systematic review guidelines. However, we performed a comprehensive literature review, and we believe that using the guidelines of the systematic review approach would not change the general message of our work. Reviewed articles strongly differ in used methods, study protocols, and studied populations, and in our opinion, a systematic review approach would not be well-suited to compare them. Moreover, to preserve this article’s compactness, we excluded papers assessing central chemosensitivity and its impact on the function of PChR.

## 10. Conclusions—Future Directions

The overactivity of PChR is a common finding among the HF population. There is little doubt that it is related to sympathoactivation and poor prognosis. In our review, we summarized all of the clinical trials conducted on human HF patients, compared their methodology and results, and presented a thorough synthesis of possible mechanisms which modulate PChR function.

With a growing number of novel therapies potentially modulating autonomic distortion in HF, the need for the assessment of PChR function within HF patients increases. The demand for establishing an optimal protocol thereof and the identification of clinical predictors for augmented PChS should be a crucial aim of further intense research. The role of pharmacological and device treatment in the function of PChR in both chronic and acute HF, as well as the verification of potential mechanisms of increased PChR activity in the human population, should be a matter of concern for further research.

## Figures and Tables

**Table 1:** Comparison of clinical studies with PChS assessment in HF patients.

Study	Method	Number of Participants	Age of Patients [Years]	LVEF [%]	Etiology of HF [%]	Treatment [%]	Peripheral Chemosensitivity to Hypoxia [L · min^−1^ · %SpO_2_^−1^]	Prevalence of Increased PChS [%]
Chua et al. (1996) [32]	Transient hypoxia;	38 HF patients 15 healthy controls	60.2 ± 8	25.7 ± 14.17	IHD 57.9 DCM 31.6 VHD 5.2 Alcoholic-CM 2.6 HTN-CM 2.6	Diuretics 100 ACE-I 92.1 Digoxin 26.3	HF: 0.707 ± 0.47 Controls: 0.293 ± 0.22	NR
Chua et al. (1996) [33]	Transient hypoxia	13 HF patients 8 healthy controls	60.5 ± 7.56	25.5 ± 15.48	IHD 53.8 DCM 46.2	Diuretics 100 ACE-I 92.3	HF: 0.572 ± 0.295 Controls: 0.232 ± 0.062	NR
Chua et al. (1997) [23]	Transient hypoxia	50 HF patients 12 healthy controls	58.7 ± 12.1	26.5 ± 13	IHD 58 DCM 34 VHD 2 Alcoholic-CM 4 HTN-CM 2	Diuretics 100 ACE-I 100 Digoxin 24	HF: 0.673 ± 0.41 Controls: 0.272 ± 0.201	40
Ponikowski et al. (1997) [30]	Transient hypoxia	26 HF patients	60 ± 8	25.6 ± 8.6	IHD 80.8 DCM 19.2	Diuretics 100 ACE-I 100 Digoxin 100 Nitrates 100	HF: 0.72 ± 0.36	42
Ponikowski et al. (1997) [67]	Transient hypoxia	14 HF patients	60 ± 1.1	26.6 ± 11.1	IHD 78.6 DCM 21.4	BB 0	HF: 0.6 ± 0.28 (mean of 9 subjects with higher PChS)	64
Chua et al. (1997) [68]	Transient hypoxia	12 HF patients	65.5 ± 5.19	21.3 ± 10.38	IHD 66.7 DCM 33.3	Diuretics 100 ACE-I 100	HF: 0.746 ± 0.36	NR
Ponikowski et al. (1999) [34]	Transient hypoxia	74 HF patients	57 ± 10	25 ± 10	IHD 77 DCM 23	ACE-I 93 Diuretics 97 Digoxin 29	HF with CSR: 0.80 ± 0.48 HF with PB: 0.75 ± 0.68 HF with NB 0.34 ± 0.16	NR
Ponikowski et al. (1999) [69]	Transient hypoxia	39 HF patients (13 with cachexia) 11 healthy controls	60 ± 9	24 ± 9	IHD 87.2 DCM 25.6	Diuretics 94.8 ACE-I 87.2 Digoxin 48.7	HF: 0.62 ± 0.34 (cachectic: 0.91 ± 0.37 non-cachectic: 0.47 ± 0.2) Controls: 0.29 ± 0.21	NR
Ponikowski et al. (2001) [70]	Transient hypoxia	38 HF patients 12 healthy controls	57.8 ± 8	26.2 ± 11.7	IHD 74 DCM 26	Diuretics 100 ACE-I 95 Digoxin 39	HF: 0.6 ± 2.46 Controls: 0.2 ± 0.35	NR
Ponikowski et al. (2001) [25]	Transient hypoxia	80 HF patients	58 ± 9	24 ± 12	IHD 69 DCM 31	ACE-I 93 Diuretics 98 Digoxin 31	HF: 0.69 ± 0.50	34
Giannoni et al. (2008) [24]	Hypoxic isocapnic rebreathing technique;	60 HF patients 12 healthy controls	66 ± 7.75	31 ± 6.98	IHD 38 Idiopathic 50 Secondary 12	Diuretics 90 BB 92 ACE-I 62 ARB 22 MRA 62	HF: 0.74 ± 0.47 Controls: 0.35 ± 0.2	40
Giannoni et al. (2009) [35]	Hypoxic isocapnic rebreathing technique	110 HF patients	62 ± 15	31.1 ± 7.1	IHD 47 Idiopathic 40 Secondary 13	Diuretics 80 BB 86 ACE-I/ARB 78 MRA 42 CRT 27 ICD 17	HF: 0.67 ± 0.45	40
Niewinski et al. (2013) [31]	Transient hypoxia	34 HF patients 16 healthy controls	62 ± 11	27 [20,21,22,23,24,25,26,27,28,29,30]	IHD 71	BB 100 ACE-I 91 MRA 88 Diuretics 74 ICD 50 CRT 29	HF: 0.58 [0.32–0.95] Controls: 0.17 [0.06–0.29]	44
Mirizzi et al. (2016) [71]	Hypoxic isocapnic rebreathing technique	191 HF patients	62 ± 14	30 ± 8	IHD 48	BB 84 ACE-I/ARB 77 MRA 56 Diuretics 79	HF: 0.5 [0.2–1.2]	34
Collins et al. (2020) [73]	Transient hypoxia	12 HF patients 12 healthy controls	53.6 ± 12.8	43.0 ± 8.7	NR	BB 83 ACE-I/ARB 100 MRA 83 Diuretics 50	HF: 0.81 ± 0.59 Controls: 0.39 ± 0.17	NR
Tubek et al. (2021) [72]	Transient hypoxia	30 HF patients 30 healthy controls	62 ± 10	27.4 ± 7	NR	BB 100 ACE-I/ARB 100 MRA 90 Diuretics 70	HF: 0.6 ± 0.4 Controls: 0.3 ± 0.2	40
Giannoni et al. (2022) [36]	Hypoxic isocapnic rebreathing technique	369 HF patients	65 ± 12	31 [25,26,27,28,29,30,31,32,33,34,35,36,37,38]	IHD 43	BB 95 ACE-I/ARB 89 ARNI 4 MRA 77 Diuretics 71 ICD 21 CRT 19	HF: 0.5 [0.3–0.9] (267 subjects)	29

Values are presented as mean ± standard deviation (SD); median [interquartile range] or percentages. ACE-I—angiotensin-converting enzyme inhibitor; ARB—angiotensin receptor blocker; ARNI—angiotensin receptor–neprilysin inhibitor; BB—beta-blocker; CM—cardiomyopathy; CRT—cardiac resynchronization therapy; CSR—Cheyne–Stokes respirations; DCM—idiopathic dilated cardiomyopathy; HF—heart failure; HTN-CM—hypertensive cardiomyopathy; ICD—implantable cardioverter-defibrillator; IHD—ischemic heart disease; LVEF—left ventricular ejection fraction; MRA—mineralocorticoid receptor antagonist; NB—normal breathing; NR—not reported; PB—periodic breathing; PChS—peripheral chemosensitivity; VHD—valvular heart disease.

**Table 2:** Comparison of clinical studies using hyperoxia in HF patients.

Study	Time of O_2_ Inhalation	Rest/Exercise	Number of Participants	Age of Patients	LVEF [%]	Etiology of HF [%]	Treatment [%]	Effects of Hyperoxia
Chua et al. (1996) [33]	3 breaths	rest; exercise	13 HF patients 8 healthy controls	60.5 ± 7.6	25.5 ± 15.5	IHD 53.8 DCM 46.2	Diuretics 100 ACE-I 92.3	↓ Ventilation (HF and Controls, *p* = NS)
Chua et al. (1996) [33]	NR	exercise	12 HF patients	65.5 ± 5.2	21.3 ± 10.4	IHD 66.7 DCM 33.3	Diuretics 100 ACE-I 100	↑ exercise time ↓ ventilatory response to exercise
Ponikowski et al. (1997) [30]	20 min	rest	12 HF patients	NR	NR	NR	NR	↑ LFr and HFr power of HRV ↑ α index
Hennersdorf et al. (2001) [74]	5 min	rest	23 HF patients 26 healthy controls	62.9 ± 7.9	29.9 ± 9.6	IHD 91 DCM 9	ACE-I 100 Digoxin 100 Diuretics 100	↓ HR (HF < Controls)
Franchitto et al. (2010) [75]	15 min	rest	18 HF + anemia patients 18 HF controls	63.4 ± 11	29.9 ± 8.9	IHD 77	BB 89 ACE-I/ARB 66 Diuretics 72	↓ MSNA (HF + anemia)
Despas et al. (2012) [29]	15 min	rest	18 HF patients with augmented chemosensitivity 20 HF controls	63.7 ± 16.1	29.5 ± 10.6	IHD 67 DCM 28 VHD 6	BB 78 ACE-I 72 Diuretics 83	↑ arterial baroreflex gain (HF with augmented PChS) ↓ MSNA (HF with augmented PChS)
Edgell et al. (2015) [27]	2 min	rest	11 HF patients 10 healthy controls	60.3 ± 10	38.7 ± 15.3	NR	BB 100 ACE-I 90.9 Diuretics 81.8	↓ HR (HF and Controls) ↓Ventilation (HF)
Tubek et al. (2021) [72]	1 min	rest	30 HF patients 30 healthy controls	62 ± 10	27.4 ± 7	no data	BB 100 ACE-I/ARB 100 MRA 90 Diuretics 70	↑ SVR, MAP (Controls) ↔ HR, MAP (HF) ↓ CO (HF and Controls) ↓ HR (Controls) ↓ Ventilation (HF)

Values are presented as mean ± standard deviation (SD) or percentages. ACE-I—angiotensin-converting enzyme inhibitor; ARB—angiotensin receptor blocker; BB—beta-blocker; CO—cardiac output; DCM—idiopathic dilated cardiomyopathy; HF—heart failure; HFr—high frequency; HR—heart rate; HRV—heart rate variability; IHD—ischemic heart disease; LFr—low frequency; LVEF—left ventricular ejection fraction; MAP—mean arterial pressure; MRA—mineralocorticoid receptor antagonist; MSNA—muscle sympathetic nerve activity; NR—not reported; NS—not significant; PChS—peripheral chemoreceptor sensitivity; SVR—systemic vascular resistance; VHD—valvular heart disease; ↑—increase; ↔—unchanged; ↓—decrease.

**Table 3:** Comparison of clinical studies using dopamine in HF patients.

Study	Dose of Dopamine [µg·kg^−1^·min^−1^]	Rest/Exercise	Number of Participants	Age of Patients	LVEF [%]	Etiology of HF [%]	Treatment [%]	Effects of Dopamine
Van der Borne et al. (1998) [28]	5	rest	8 HF patients 8 healthy controls	57 ± 12	NR	IHD 62.5 Idiopathic 37.5	NR	↔ MAP, HR ↓ MV, HVR
Collins et al. (2020) [73]	2	exercise	12 HF patients 12 healthy controls	53.6 ± 12.8	43.0 ± 8.7	NR	BB 83 ACE-I/ARB 100 MRA 83 Diuretics 50	↑ P_ET_CO_2_, CO, SV, CO/MAP ↔ exercise time, MAP, HR
Edgell et al. (2015) [27]	2	rest	11 HF patients 10 healthy controls	60.3 ± 10	38.7 ± 15.3	NR	BB 100 ACE-I 90.9 Diuretics 81.8	↑ CO, SV ↔ HR, MAP ↓ MV

Values are presented as mean ± standard deviation (SD) or percentages. ACE-I—angiotensin-converting enzyme inhibitor; ARB—angiotensin receptor blocker; BB—beta-blocker; CO—cardiac output; CO/MAP—conductance; HF—heart failure; HR—heart rate; HVR—hypoxic ventilator response; IHD—ischemic heart disease; LVEF—left ventricular ejection fraction; MAP—mean arterial pressure; MRA—mineralocorticoid receptor antagonist; MV—minute ventilation; NR—not reported; PETCO_2_—end-tidal partial pressure of carbon dioxide; SV—stroke volume; ↑—increase; ↔—unchanged; ↓—decrease.

**Table 4:** Predictors of increased chemosensitivity extracted from clinical trials.

Group of Predictors	Characteristics of Patients with High Chemosensitivity
Biochemical	↓Hb [75] ↑GGT [71] ↑NA [24,36,71] ↑NT-proBNP [24,31,36,71] ↑BNP [24]
Clinical	Cardiac cachexia [69] ↑NYHA class [23,24,35,70,74] ↑Age [71] ↑MSNA [29] ↓Arterial baroreflex sensitivity [29] ↓Renal function [36]
Haemodynamic	↓LVEF [23,25,31,70,71,74] ↑nsVT [23,35,74] ↑AF [35] ↓HRV [31] ↑RV dimensions [71] ↑SBP [31] ↑Systolic pulmonary pressure [36]
Respiratory	↓Peak VO_2_ [31,35,70] ↑VE/VCO_2_ slope [23,35,36,70,71]

AF—atrial fibrillation; BNP—B-type natriuretic peptide; GGT—gamma-glutamyltransferase; Hb—hemoglobin; LVEF—left ventricular ejection fraction; MSNA—muscle sympathetic nerve activity; NA—noradrenaline; nsVT—nonsustained ventricular tachycardia; NT-proBNP—N-terminal pro-B-type natriuretic peptide; RV—right ventricular; SBP—systolic blood pressure; VE/VCO_2_ slope—regression slope relating minute ventilation to carbon dioxide output; VO_2_—oxygen consumption during exercise; ↑—increase; ↓—decrease.

## Data Availability

Not applicable.
